# Effect of medication therapy combined with transcranial direct current stimulation on depression and response inhibition of patients with bipolar disorder type I: a clinical trial

**DOI:** 10.1186/s12888-021-03592-6

**Published:** 2021-11-17

**Authors:** Parnaz Mardani, Ahmad Zolghadriha, Mohsen Dadashi, Hossein Javdani, Seyedeh Elnaz Mousavi

**Affiliations:** 1 Department of Clinical Psychology, School of Medicine, Zanjan University of Medical Sciences, Zanjan, Iran; 2 Department of Psychiatry, School of Medicine, Zanjan University of Medical Sciences, Zanjan, Iran; 3 Department of Psychiatry, School of Medicine, Qazvin University of Medical Sciences, Qazvin, Iran; 4 Department of Clinical Psychology, School of Medicine, Shahid Beheshti University of Medical Sciences, Tehran, Iran

**Keywords:** Bipolar disorder, Transcranial direct current stimulation, Medication, Depression, Response inhibition

## Abstract

**Objective:**

Bipolar Disorder (BD) is one of the most common mental disorders associated with depressive symptoms and impairment in executive functions such as response inhibition. This study aimed to investigate the effectiveness of medication therapy combined with Transcranial Direct Current Stimulation (tDCS) on depression and response inhibition of patients with BD.

**Method:**

This is a double-blinded randomized clinical trial with pretest, posttest, and follow-up design. Participants were 30 patients with BD randomly assigned to two groups of Medication+tDCS (*n* = 15, receiving medications plus tDCS with 2 mA intensity over dorsolateral prefrontal cortex for 10 days, two sessions per day each for 20 min) and Medication (*n* = 15, receiving mood stabilizers including 2–5 tables of 300 mg (mg) lithium, 200 mg sodium valproate, and 200 mg carbamazepine two times per day). Pretest, posttest and 3-month follow-up assessments were the 21-item Hamilton Depression Rating Scale (HDRS) and a Go/No-Go test. Collected data were analyzed in SPSS v.20 software.

**Results:**

The mean HDRS score in both groups was reduced after both interventional techniques, where the group received combined therapy showed more reduction (*P* < 0.01), although their effects were not maintained after 3 months. In examining response inhibition variable, only the combined therapy could reduce the commission error of patients under a go/no-go task (*p* < 0.05), but its effect was not maintained after 3 months. There was no significant difference in the group received medication therapy alone.

**Conclusion:**

Medication in combination with tDCS can reduce the depressive symptoms and improve the response inhibition ability of people with BD.

**Trial registration:**

This study was registred by Iranian Registry of Clinical Trials (Parallel, ID: IRCT20191229045931N1, Registration date: 24/08/2020).

## Introduction

Bipolar Disorder (BD) is a common psychological disorder that affects about 1–5% of the total population [1]. It is associated with significant impairment in work, family, and social life [2]. General symptoms of BD are mood disturbances and emotional dysregulation which can lead to impairment in mood stability and executive functioning [3, 4]. There are two main types of bipolar disorders: bipolar I and bipolar II. According to the Diagnostic and Statistical Manual of Mental Disorders-Fifth edition (DSM-5) [5], bipolar I disorder involves episodes of severe mania and often depression. During a manic episode, elevated mood can manifest itself as either euphoria or as irritability. People in manic episodes may spend more money or pursue unrealistic plans. Depressive episodes in bipolar disorder are similar to regular clinical depression, with depressed mood, loss of pleasure, low energy and activity, feelings of guilt or worthlessness, and thoughts of suicide. Bipolar II disorder involves a less severe form of mania called hypomania. Epidemiological studies have reported a prevalence of 0.6% for bipolar I and 0.4% for bipolar II in the world [6], while in Iran it is 0.04% for bipolar I and 0.3% for bipolar II [7]. There is evidence of cognitive impairment in BD patients [8–12]. One of the most important cognitive processes that can be impaired in BD patients is response inhibition or inhibitory control [13]. It is an ability that helps a person to stop and think before acting and decide when to respond. It is the ability to inhibit or control impulsive (or automatic) responses, and create responses by using attention and reasoning.

Different methods have been used to treat BD. One of these methods is medication therapy; however, with the onset of symptoms and recurrence of the disorder, patients may need hospital admission and then begin a new cycle of the medication process. Side effects, patients’ resistance to medication, and restrictions on medication use in some patients are among the disadvantages of medication. Therefore, there is a need for a safe and more effective method with fewer side effects. Recent technological advances in non-invasive brain stimulation have opened new perspectives in the treatment of psychiatric disorders. One of these methods is transcranial Direct Current Stimulation (tDCS). As a non-invasive technique of neuromodulation, it can modulate the cortical excitability by applying a weak electrical current (2 mA) over the scalp through two electrode surfaces (one anode and one cathode). Anodal tDCS causes a depolarization of neurons and thus increases cortical excitability, while cathodal tDCS causes neuronal hyperpolarization and reduces cortical excitability [14, 15]. Studies have shown that tDCS is effective in treating major depressive disorder [16–21] and response inhibition [22, 23]. Neurobiologically, the prefrontal cortex, especially Dorsolateral Prefrontal Cortex (DLPFC), is a critical region in the cognitive control of behaviors [24]. In this regard, most of previous studies have applied tDCS over this cortical area (e.g. [24, 25]). The use of tDCS can be a good treatment option for patients who experience many side effects after taking medications or for patients who are resistant to medication treatment [26]. To our knowledge, no clinical trial has examined the effectiveness of tDCS combined with medication in improvement of cognitive and psychological disorders in BD patients. In this regard, this study aimed to evaluate the effect of medication therapy combined with tDCS on depression and response inhibition of patients with BD.

## Methodology

### Study desig and participants

This study is a double-blinded randomized clinical trial (Parallel, ID: IRCT20191229045931N1; registration date: 24/08/2020; http://en.irct.ir/trial/45956) with a pretest/posttest/follow-up design conducted in April 2020 (during the COVID-19 pandemic). The study population consists of all outpatients with BD type I referred to Sohravardi General Outpatient Clinic belonged to Shahid Beheshti Hospital in Zanjan, Iran. The sample size was determined 15 for each group using Gpower software considering α = 0.05 and an effect size of 0.6. Therefore, 30 depressed ones were selected by using a purposive sampling method and based on the inclusion criteria (willingness to participate, having BD type 1 diagnosed by a psychiatrist, having depreesion according to the Hamilton Depression Rating Scale (HDRS) score, age 18–50 years, at least a middle school education, no severe psychiatric disorders such as psychotic disorders and cognitive impairment, no history of epileptic seizures and head injuries, no substance and alcohol use, and receiving no psychological and technological intervention at least 1 month before study). Absence from more than two intervention sessions, suicidal ideation, need for electroconvulsive therapy during the intervention, unwillingness to continue participation, presence of metal or electrical device in the head, and pregnancy were the criteria for exclusion from the study. Participants were assigned into two groups of medication (*n* = 15) and medication + tDCS (*n* = 15) using Random Number Generator program.

### Measures

After obtaining written informed consent from the patients for participating in the study, their demographic data (age, gender, education, marital status) were recorded. Then, they completed the 21-item HDRS and underwent Go/No-Go test. The HDRS, developed by Hamilton [27], was used to assess the severity of depressive symptoms in patients by a clinical interview. The items of HDRS are scored from 0 to 2 or 0–4 measuring depressed mood, feelings of guilt, suicide, initial insomnia, insomnia during the night, delayed insomnia, work and interests, retardation, agitation, psychiatric anxiety, somatic anxiety, gastrointestinal somatic symptoms, general somatic symptoms, genital symptoms, hypochondriasis, weight loss, and insight. A total score of 0–7 is considered normal, while scores of 20 or higher indicate severe depression. Its sensitivity and specificity in diagnosing positive cases of depression are 86 and 92%, respectively [28]. For its Persian version, the validity through evaluating its correlation with Beck Depression Inventory and Dysfunctional Attitude Scale has been reported r = 0.55 and r = 0.39, respectively, with reported inter-rater reliability of 0.95 [29].

The Go/No-Go test was used to assess response inhibition/ inhibitory control of patients. To perform this computerized test, pairs of rectangles (white/green and white/yellow) appeared randomly on the screen for a short time. If one of these pairs was yellow, the patient was asked to make no response, but if one of them was green, s/he was asked to give one of the two following responses: If the green rectangle was on the right and the white rectangle on the left, press the keyboard button “/” rapidly, but if the white rectangle was on the right and the green rectangle on the left, press the “z” button rapidly. In the end, their commission error rate, omission error rate, inhibition, and response time (ms) were recorded. Fewer errors indicate a better response.

### Interventions

Afterwards, the medication group received mood stabilizers including 2–5 tablets of 300 mg lithium (dosage ranged from 600 to 1500 mg), 200 mg sodium valproate (dosage ranged from 400 to 1500 mg), and 200 mg carbamazepine (dosage ranged from 400 to 800 mg), two times per day, while the second groups received both medication therapy and tDCS intervention. The tDCS (2-mA intensity for 20 min with a ramp-like fade-in time of 15 s and fade-out time of 30 s) was performed using a FDA-approved device (Oasis Pro, Mind Alive Inc., Canada) for 10 consecutive days, two sessions per day. The number of sessions was determined according to De Almeida et al. [30]. Two electrodes (positive anode and negative cathode) covered by a sponge soaked in saline were positioned in the subjects’ head over right DLPFC (anode position over F3 and cathode over F4 according to the EEG 10–20 International System). Immediately and 3 months after the intervention, patients completed HDRS questionnaire and performed Go/No-Go test again. It should be mentioned that assessments before and after intervention were performed by another expert who was unaware of the results.

### Statistical analysis

Collected data were analyzed in SPSS v.20 using descriptive statistics (frequency, percentage, mean, and standard deviation), and statistical tests including ANCOVA, multivariate and univariate ANOVA, independent t-test, and chi-square test. Before conducting these tests, the normality of data distribution was reported by Kolmogorov-Smirnov test (*p* > 0.05) and the equality of variances was reported by Levene’s test (*p* > 0.05).

## Results

Table 1 presents the descriptive statistics for the demographic characteristics of participants. Of 30 participants, 10 were male and 5 females in the medication group where most of them were single with a high school diploma (mean age = 30.33 ± 8.63 years), while there were 3 male and 12 females in the combined therapy group where most of them were married with a high school diploma (mean age = 32.06 ± 9.43 years). At baseline, independent t-test results showed no significant difference between the two groups in terms of age (*P* > 0.05), while chi-square test indicated a statistically significant difference in terms of gender (*p* < 0.05), but not in terms of educational level and marital status (Table 1).

**Table 1:** Demographic characteristics of participants in two study groups

Demographic factors		Medication (***n*** = 15)		Medication + tDCS (***n*** = 15)		***P***-value*
		N	%	N	%	
Gender	Male	10	66.7	3	20	0.01
	Female	5	33.3	12	80	
Marital status	Married	7	46.7	8	53.3	0.71
	Single	8	53.3	7	46.7	
Educational level	Lower than high school	2	13	2	13.3	0.90
	High school diploma	9	60	10	66.7	
	Bachelor’s degree	4	26.7	3	20	
		**Mean ± SD**		**Mean ± SD**		*P*-value**
Age		30.33 ± 8.63		32.06 ± 9.43		0.60

* Chi-square test; ** independent t-test

The mean HDRS scores of patients at three evaluation phases are presented in Table 2. As can be seen, the depression of patients in both groups was reduced after intervention, where the group received both medication and tDCS showed more reduction (21.40 ± 8.10 vs. 1.80 ± 2.21) than the group received medication alone (15.60 ± 11.28 vs. 13.53 ± 22.02). In both groups, the decrease continued slightly in the follow-up period. According to ANCOVA results shown in Table 3, after controlling covariate (pretest score), this difference between groups was statistically significant (F (1,27) = 50.20, *p* = 0.000 < 0.05) and it can be said that the combination of medication with tDCS had a higher impact on the depression of BD patients. The obtained effect size (η^2^) indicated that 65% of the changes in the groups were due to the effect of the combined intervention. To measure the effect of the time factor, within-subject comparison by using repeated-measures ANOVA was conducted (Table 4). The results revealed an overall significant difference between the means at three different time points of pretest, posttest, and follow-up (*p* = 0.000 < 0.05). Pairwise comparison using Bonferroni test (Table 5) showed that, in combined therapy group, there was a significant difference between pretest and posttest HDRS scores (*p* = 0.000), and between pretest and follow-up HDRS scores (*p* = 0.000), but no significant differences between posttest and follow-up scores (*p* > 0.05).

**Table 2:** Mean scores of HDRS and Go/No-Go test in two study groups

Variables	Time	Groups	
		Medication, Mean ± SD	Medication + tDCS, Mean ± SD
Depression	Pretest	15.60 ± 11.28	21.40 ± 8.10
	Posttest	13.53 ± 22.02	1.80 ± 2.21
	Follow-up	11.93 ± 8.38	1.46 ± 2.32
Commission error	Pretest	1.86 ± 2.58	2.26 ± 1.98
	Posttest	2.46 ± 3.46	0.73 ± 1.27
	Follow-up	1.46 ± 3.54	0.93 ± 1.57
Omission error	Pretest	0.33 ± 0.72	3.46 ± 7.98
	Posttest	3.06 ± 8.09	0.06 ± 0.25
	Follow-up	1.60 ± 5.92	1.53 ± 5.93
Average response time (ms)	Pretest	369.46 ± 46.06	405.40 ± 111.29
	Posttest	387.33 ± 78.60	370.53 ± 70.27
	Follow-up	373.20 ± 60.86	382.20 ± 73.86

*SD* Standard deviation

**Table 3:** Test of between-subject effects (dependent variable: posttest depression)

Source	Sum of Squares	df	Mean Square	F	***P*** value	Partial Eta squared
Corrected model	1794.25	2	874.62	29.54	0.000	0.68
Intercept	21.68	1	21.68	0.73	0.400	0.02
Pretest depression	716.71	1	716.71	24.20	0.000	0.47
Group	1486.45	1	1486.45	50.20	0.000	0.65
Error	799.41	27	29.60	–	–	–
Total	4312	30	–	–	–	–

**Table 4:** Test of within-subject effects for depression variable (Greenhouse-Geisser test)

Source	Sum of Squares	df	Mean Square	F	Sig.	Partial Eta squared	Test Power
Depression	2575.35	1.55	1658.59	62.34	0.000	0.69	1
Depression*Group	1434.06	1.55	923.57	34.71	0.000	0.55	1
Error	1156.57	43.47	26.602	–	–	–	

* Indicates the interaction effect of time and group

**Table 5:** Pairwise comparison for the depression variable

Group	(I) time	(J)time	Mean Difference (I-J)	Std. Error	Sig.	95% CI	
						Lower bound	Upper bound
tDCS+ medication	Pretest	Posttest	19.60^a^	1.93	0.000	14.33	24.87
		Follow-up	19.93^a^	1.93	0.000	14.67	25.18
	Posttest	Pretest	−19.60^a^	1.93	0.000	−24.87	− 14.33
		Follow-up	0.33	0.31	0.940	−0.53	1.20
	Follow-up	Pretest	−19.93^a^	1.93	0.000	−25.18	−14.67
		Posttest	−0.33	0.31	0.940	−1.20	0.53
Medication	Pretest	Posttest	2.06	1.75	0.774	−2.69	6.82
		Follow-up	3.66	1.84	0.200	−1.34	8.67
	Posttest	Pretest	−2.06	1.75	0.774	−6.83	2.69
		Follow-up	1.60	1.56	0.974	−2.66	5.86
	Follow-up	Pretest	−3.66	1.84	0.200	−8.67	1.34
		Posttest	−1.60	1.56	0.974	−5.86	2.66

^a^The mean difference is significant at *p* < 0.05

Regarding the mean scores of Go/No-Go test shown in Table 2, it can be seen that when we used medication alone, their errors and average response time increased, while the combination of medication with tDCS method reduced their errors and average response time to stimuli. After controlling covariate (pretest response time), the ANCOVA results (Table 6) showed a significant difference between groups only in terms of commission error (F = 5.36, *p* = 0.02 < 0.05) indicating that the combination of medication with tDCS could improve the response inhibition of BD patients compared to when only medication was used. However, within-subject comparison (Table 7) showed no overall significant difference between the mean scores at three evaluation times (*p* > 0.05).

**Table 6:** Test of between-subject effects for the Go/No-Go test dimensions

Source	Dependent Variable	Sum of Squares	df	Mean Square	F	Sig.	Partial Eta squared
Corrected model	Commission error	108.28	5	21.65	4.95	0.00	0.50
	Omission error	82.15	5	16.43	0.43	0.81	0.08
	Average response time (ms)	62,919.51	5	12,583.90	3.18	0.02	0.39
Intercept	Commission error	7.35	1	7.35	1.68	0.20	0.06
	Omission error	6.41	1	6.41	0.17	0.68	0.00
	Average response time (ms)	13,824.83	1	13,824.83	3.49	0.07	0.12
Pretest (response time)	Commission error	0.74	1	0.74	0.17	0.68	0.00
	Omission error	6.27	1	6.27	0.16	0.68	0.00
	Average response time (ms)	52,031.53	1	52,031.53	13.16	0.00	0.35
Group	Commission error	23.45	1	23.45	5.36	0.02	0.18
	Omission error	63.30	1	63.30	1.68	0.20	0.06
	Average response time (ms)	3292.17	1	3292.17	0.83	0.37	0.03
Error	Commission error	104.91	24	4.37			
	Omission error	903.21	24	37.63			
	Average response time (ms)	94,844.35	24	3951.84			
Total	Commission error	290	30				
	Omission error	1059	30				
	Average response time (ms)	4,465,478	30

**Table 7:** Test of within-subject effects for inhibitory control variable (Greenhouse-Geisser test)

Source	Sum of Squares	df	Mean Square	F	Sig.	Partial Eta squared	Test Power
Commission error	23,143.08	1.16	19,867.04	279.52	0.000	0.90	1
Commission error*Group	172.02	1.16	147.67	2.07	0.15	0.69	0.30
Error	2318.22	32.61	71.074				
Omission error	2,977,604.46	1.00	2,957,508.54	612.17	0.000	0.95	1
Omission error*Group	7340.46	1.00	7290.92	1.50	0.23	0.05	0.22
Error	136,191.06	28.19	4831.140				
Average response time	2,605,127.46	1.08	2,410,601.21	824.31	0.000	0.96	1
Average response time*Group	331.02	1.08	306.30	0.10	0.76	0.00	0.06
Error	88,490.17	30.25	2924.377				

* Indicates the interaction effect of time and group

## Discussion

TDCS is a non-invasive brain stimulation technique that modulates cortical excitability and spontaneous brain activity in a safe, economic, and well-tolerated manner. Since BD is a common and complicated disorder that sometimes causes long-term use of psychiatric medications, the use of new alternative therapies such as TDCS can be effective in improving the performance of BD patients. To the best of our knowledge, this is the first clinical trial that investigates the effect of tDCS combined with medication therapy on depression and inhibitory control of patients with BD. The findings of our study revealed the effectiveness of right anode/left cathode tDCS (2 mA, 20 min) combined with medication in reducing depression of adults with BD compared to those received medication only. Some studies have examined the effect of tDCS on the depression of BD patients. In a clinical trial by Sampaio-Junior et al. [16], patients with bipolar depression received left prefrontal anodal stimulation as an add-on treatment to their pharmacological therapy. Patients receiving active stimulation had a more significant symptom reduction as compared to those treated with sham tDCS. Dondé et al. [31] in a meta-analysis showed that tDCS could improve depressive symptoms in patients with bipolar depression, particularly after 1 week of treatment. Herrera-Melendez et al. [32] in a review study also found out that tDCS potentially improves depressive symptoms in patients with bipolar depression. Aparicio et al. [18] and Martin et al. [33] also concluded that tDCS can reduce depressive symptoms. McClintock et al. [20] in a controlled clinical trial, concluded that tDCS could have positive neurocognitive effects in patients with unipolar and bipolar depression. There is one published case report on the combination of tDCS with pharmacological treatment in a male patient with an acute episode of mania [34] where the authors performed anodal tDCS over the right DLPFC combined with a pharmacological intervention and reported an improvement of manic symptoms that lasted until 72 h after stimulation. These findings are consistent with our results. In our study, the use of medication alone (mood stabilizers) also reduced depression of BD patients which is consistent with the results of Sachs et al. [35] and Pacchiarotti et al. [36], who reported the effect of antidepressant drugs in BD patients.

The DLPFC has been linked to depression due to decreased left DLPFC performance and increased right DLPFC performance [37]. During the depression, there is a possibility of dysfunction along with decreased regional blood flow or glucose metabolism in left DLPFC [38] and hyperactivity of the right DLPFC (according to the theory of prefrontal asymmetry), The right anode/left cathode tDCS can therefore help reduce depressive symptoms [37]. On the other hand, although mood stabilizers have been approved by the FDA for treating patients with BD, they are not enough because some patients are resistant to these medications or high doses of these medications can reduce the patients’ daily performance. The use of tDCS can facilitate the effects of medication therapy. It can modulate synaptic transmission by regulating the dose of transmitters, including serotonin [39]. Hence, it is suggested that tDCS can be a useful method for treating depression of BD patients combined with medication. The effect of combined therapy in our study was not stable for 3 months after stimulation. This may be because of the low number of sessions and duration (10 sessions each for 20 min). The use of high number and longer sessions may affect the stability of its effect.

The findings of our study showed the effectiveness of combining medication therapy with tDCS in reducing commission error of BD patients under a go/no-go task compared to the medication therapy alone, indicating its impact on the improvement of their response inhibition ability. Some studies have reported the effect of tDCS on inhibitory control such as Hogeveen eta al [22]., Cunillera et al. [40], and Wynn et al. [41], and reported that the placement of electrodes in different areas of the brain causes different benefits over time. For explaining the results, it can be said that the frontal-lateral region, as one of the important regions of the prefrontal cortex which was simulated in our study, is responsible for identifying and determining actions, evaluating and predicting the consequences of current behavior, social control, and response inhibition. Moreover, based on the Barkley’s inhibition model, it is assumed that the proper executive functioning depends on the proper functioning of inhibition in the frontal and prefrontal cortex [42]. Furthermore, it has been reported that the F3 region in the prefrontal cortex plays an important role in inhibiting inappropriate behavioral responses [43]. Therefore, stimulation of this area could improve the management of impulsive behaviors and response inhibition in BD patients by regulating the activity of frontal, prefrontal lobes, and anterior cingulate cortex. As mentioned above, tDCS modulates synaptic transmission by regulating the dose of transmitters. Hence, it can be said that TDCS is a complementary therapy and can accelerate and improve the mechanism of medication therapy.

In our study, gains in response inhibition ability were not maintained 3 months after intervention. Stable results may be achieved if the number and duration of tDCS sessions increases or the stimulated area is changed. Since BD is one of the most severe psychiatric disorders and, on the other hand, response inhibition is severely impaired in these patients [44] and is associated with other behavioral consequences, other interventions such as emotion regulation strategies and cognitive rehabilitation are required along with tDCS to stabilize the increased response inhibition. Medication therapy alone (use of mood stabilizers) did not improve response inhibition ability of patients in our study. This is consistent with the results of Pavuluri et al. [45] who showed that treatment with either risperidone or divalproex failed to dampen disordered amygdala connectivity in the occipital-limbic network during a response inhibition fMRI task, but is against the results of Pavuluri et al. [46] who showed that treatment with second-generation antipsychotics followed by lamotrigine monotherapy enhanced prefrontal and temporal lobe activity during a go/no-go task.

Some of the limitations of the present study included: Use of a purposive sampling method, small sample size (Lack of BD patients in the outpatient clinic due to the COVID-19 pandemic), lack of cooperation of male patients, no sham tDCS group (Due to difficulty recruiting patients during the COVID-19 pandemic), and not examining different tDCS protocols. Therefore, it is suggested that further studies be performed using a larger sample size and objective tools such as electroencephalography or functional magnetic resonance imaging. Moreover, it is recommended that the effect of combining medication therapy with other novel therapies such as neurofeedback or repetitive Transcranial Magnetic Stimulation be investigated.

## Conclusion

Combination of tDCS with medication therapy can significantly reduce depressive symptoms and improve response inhibition ability of adults with BD in a short period.

## Data Availability

The datasets used and analysed during the current study are available from the corresponding author on reasonable request.
